# Radiofrequency Catheter Ablation of Ventricular Premature Complexes

**Published:** 2010-08-15

**Authors:** Johnson Francis

**Affiliations:** 1 Professor of Cardiology, KMCT Medical College, Calicut, Kerala, India; 2 Visiting Consultant, Malabar Institute of Medical Sciences, Calicut, Kerala, India

**Keywords:** ventricular premature complexes, catheter ablation

Frequent ventricular premature complexes  (VPC) can result in left ventricular dysfunction. Several case reports have found this association and reversal with radiofrequency ablation [1-4]. Other peculiar problems reported to be due to VPCs and relieved by radiofrequency ablation include intermittent claudication [5], chronic cough with cough syncope [6,7] and dysphagia [8], mostly by the same group of authors. An interesting case in which radiofrequency catheter ablation of VPCs resulted in improvement of left ventricular function in a non-responder to cardiac resynchronization therapy has also been reported [9].

The relation between the burden of ventricular ectopy and left ventricular function was evaluated by Baman TS et al [10]. VPC burden was estimated by 24-hour Holter monitoring in 174 patients referred for VPC ablation. Receiver-operator characteristic curves were used to determine a cutoff VPC burden associated with left ventricular dysfunction. Reduced left ventricular ejection fraction was seen in one third of the patients and they had an average VPC burden of 33 ± 13% while the VPC burden in those with normal left ventricular function was 13 ± 12% (p < 0.0001). A VPC burden of >24% was independently associated with reversible VPC-induced cardiomyopathy. Higher number of VPCs per day was associated with larger left ventricular size and more depressed systolic and diastolic function in the report by Lelakowski J, et al [11] as well. In a retrospective analysis of 108 patients with VPCs originating from right ventricular outflow tract, the prevalence of left ventricular dysfunction was 4%, 12% and 34% respectively in those with <1000, 1000-10,000 and >10,000 VPCs per 24 hours [12].

Improvement in left ventricular end systolic and end diastolic dimensions as well as ejection fraction has been documented after VPC ablation [11]. In spite of the fact that those with depressed global ejection fraction (<50%) were excluded from this study involving 22 patients, almost 10% improvement in left ventricular ejection fraction was noted in this study (p < 0.001).  There was also improvement in NYHA functional class and exercise capacity with ablation.  Normalization of clinical status and left ventricular systolic function and dimensions have been noted regardless of the whether the VPCs were originating from the right or left ventricle [13], though it may be logical to assume that improvement may occur only in case of VPCs originating from the right ventricular outflow tract. Bogun F, et al [14] found that while left ventricular function normalized over a period of 6 months in 18 patients in whom ablation was successful, ejection fraction declined further in 4 patients in whom ablation was ineffective. The improvement noted was from a baseline of 34% to 59 ± 7%.

Darrieux FC, et al [15] noted an initial success of 76.6% with radiofrequency ablation of VPCs originating from the right ventricular outflow tract among 30 consecutive patients. Of the 9 patients who underwent a second procedure (7 initial failures and 2 with recurrence) 5 were successful, making up a final success rate of 80%.

Lelakowski J et al [16] reported improved quality of life in 22 patients who underwent VPC ablation. They also observed a negative correlation between VPC load and quality of life.

Two articles in this issue of the journal focus on the ablation of VPCs. While Sheldon SH et al [17] discusses the association with sudden cardiac death, risk stratification and management, Mokabberi R et al [18] reports transient global amnesia after ablation of VPCs arising from the right coronary cusp.
